# Economic savings for scientific free and open source technology: A review

**DOI:** 10.1016/j.ohx.2020.e00139

**Published:** 2020-09-09

**Authors:** Joshua M. Pearce

**Affiliations:** a Department of Materials Science and Engineering, Michigan Technological University, Houghton, MI 49931, USA; b Department of Electrical and Computer Engineering, Michigan Technological University, Houghton, MI 49931, USA; c Department of Electronics and Nanoengineering, School of Electrical Engineering, Aalto University, Espoo, Finland

**Keywords:** Open source, Open source hardware, Libre hardware, Free and open source, Open hardware, Free and open source software, Open science, Open innovation, RepRap, 3-D printing, Customization, Open science hardware, OScH, FOSS, FOSH, Custom designs, Distributed manufacturing, P2P, P2P manufacturing, Open design, Scientific equipment, Open scientific hardware, Instrumentation, Scientific instruments, Science funding, Science policy, Economics, Science finance, Science

## Abstract

Both the free and open source software (FOSS) as well as the distributed digital manufacturing of free and open source hardware (FOSH) has shown particular promise among scientists for developing custom scientific tools. Early research found substantial economic savings for these technologies, but as the open source design paradigm has grown by orders of magnitude it is possible that the savings observed in the early work was isolated to special cases. Today there are examples of open source technology for science in the vast majority of disciplines and several resources dedicated specifically to publishing them. Do the tremendous economic savings observed earlier hold today? To answer that question, this study evaluates free and open source technologies in the two repositories compared to proprietary functionally-equivalent tools as a function of their use of Arduino-based electronics, RepRap-class 3-D printing, as well as the combination of the two. The results of the review find overwhelming evidence for a wide range of scientific tools, that open source technologies provide economic savings of 87% compared to equivalent or lesser proprietary tools. These economic savings increased slightly to 89% for those that used Arduino technology and even more to 92% for those that used RepRap-class 3-D printing. Combining both Arduino and 3-D printing the savings averaged 94% for free and open source tools over commercial equivalents. The results provide strong evidence for financial support of open source hardware and software development for the sciences. Given the overwhelming economic advantages of free and open source technologies, it appears financially responsible to divert funding of proprietary scientific tools and their development in favor of FOSH. Policies were outlined that provide nations with a template for strategically harvesting the opportunities provided by the free and open source paradigm.

## Graphical abstract

**Figure:**
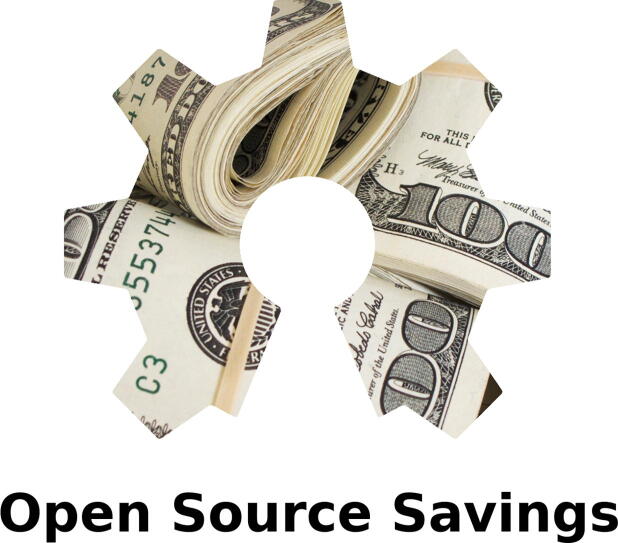

## 1 Introduction

Distributed digital manufacturing of free and open source hardware (FOSH) has shown particular promise among scientists for developing custom scientific tools [1], [2]. FOSH provides the “code” for hardware including the bill of materials, schematics, instructions, CAD designs, and other information needed to recreate a physical artifact. The growth of FOSH within academia has been rapid in the last decade and appears to be tracking the rise of free and open source software (FOSS) with about a 20-year lag [3]. Specifically, the growth of articles on FOSH indexed by Google Scholar are following an exponential increase roughly 20 years behind the same growth observed in FOSS [3]. Four advantages of free and open source technologies over traditional product design have been identified by Baden et al. [4]: 1) designs are not only free, but are developed by users, which ensure suitability for a given scientific task; 2) building your own experimental equipment yields a much deeper understanding of the principles underlying its design and a better awareness of its limits, which leads to better science; 3) manufacturing is immediate and local, which empowers laboratories all over the world, and 4) the open source movement is a global phenomenon, which recruits talented builders/makers and software coders from outside the traditional scientific establishment to help design and fabricate superior devices. The disadvantages of FOSH is 1) there are not FOSH equivalents for all proprietary scientific hardware, 2) not all scientific groups have the ability to manufacture self-built devices, 3) quality assurance and reliability are substantial challenges for self-built FOSH. Although, the primary limitations to FOSH are overcome by commercialization of FOSH (e.g. quality assurance, warranties), early studies indicated there was a substantial cost savings for self-fabricating equipment for scientists that ranged from 90 to 99% [2], [5] and applied to both standard [6] as well as specialized custom equipment [7]. As the open source design paradigm [8] has been applied in most areas of the sciences and engineering it is clear that the value of the distributed technology [9] provided a substantial return on investment for a wide array of technologies in the last decade [10].

It is possible that the savings observed in the early scientific open source technologies was isolated to special cases where the market had simply left opportunities or been isolated in the ‘low hanging fruit’. In addition, Xing found that the presence of FOSS can lead to decreases in software prices and profits for proprietary software vendors, while consumer surpluses and social welfare were increased [11]. If FOSH is reaching a similar level of maturity the same effect may be occurring, which would decrease the average savings. Today there are examples of open hardware for science in the vast majority of disciplines and several resources dedicated specifically to publishing them. Do the tremendous economic savings hold currently? To answer that question, this study will first evaluate the FOSH in two databases: *HardwareX*, a journal dedicated to FOSH and *PLOS Open Source Toolkit* that houses curated FOSH articles from *PLOS One* as well as a wide range of specialty journals. There are two enabling innovations, which provide scientists and engineers this new method of distributed digital manufacturing and were reported to provide the > 90% savings [1], [2]: 1) open source electronics like the Arduino prototyping platform (www.arduino.cc) [12] and the self-replicating rapid prototyper (RepRap) project that provides open source 3-D printing (reprap.org) [13], [14], [15]. The Arduino and associated electronics are useful for automation of a wide range of scientific equipment [2], [12]. While the RepRap 3-D printer technology can make bespoke mechanical components for developing tool libraries for optics [16] or syringe pumps [17] as well as becoming scientific tools themselves in the form of microfluidics prototypers [18], chemical handling systems [19] and 3-D microscopes [20]. The journal articles in the two repositories will be evaluated for savings over proprietary functionally-equivalent tools as a function of their use of Arduino-based electronics, RepRap-class 3-D printing, as well as the combination of the two. The results will be discussed in the context of science policy to best reduce the costs of science for the benefit of society.

## 2 Methods

Two selected databases of free and open source scientific hardware were evaluated in December 2019: *HardwareX* and the *PLOS Open Source Toolkit*. *HardwareX* is a peer-reviewed open access scientific journal published by Elsevier dedicated to the open source design and construction of scientific instrumentation. The *PLOS Open Source Toolkit (channels.plos.org/open-source-toolkit)* is a global forum for open source hardware and software research and applications and contains a collection of FOSH curated by a team of open source leaders: Tom Baden, André Maia Chagas, Jenny Molloy, Nikoleta E. Glynatsi, and Yo Yehudi. These two sources of FOSH were used because all of the articles were prescreened by the editors for open source licenses. Scientific tool designs published in the technology-specific literature is often not fully open source or lacks licensing information.

All replicants were eliminated (e.g. when *HardwareX* articles were listed in the *Toolkit* library) as well as entrants in the *Toolkit* library that did not cover a specific hardware device or were not published in the peer reviewed literature.

In total 119 articles were processed (86 in *HardwareX* and 33 in the *PLOS Open Source Toolkit*) as shown in Table A1. The devices discussed in the articles were characterized as those that used 3-D printing or an open source electronics Arduino. The material costs of the devices were recorded along with the costs of proprietary equivalents if they were available.

**Table A1:** Open source scientific tools evaluated by use of 3-D printing and Arduino, cost for open source and proprietary equivalents and percent savings as a function of all, 3-D printing, Arduino and both. (n = not provided).

	**Uses**		**Cost (USD)**		**Percent Savings (%)**			
**Title**	**3DP**	**Arduino**	**OS**	**Proprietary**	**Total**	**Only 3DP**	**Only Ard.**	**3DP + Ard.**
Lau SK, Ribeiro FA, Subbiah J, Calkins CR. Agenator: An open source computer-controlled dry aging system for beef. HardwareX. 2019;6.	0	1	$620	n				
Fortune BC, Pretty CG, Chatfield LT, McKenzie LR, Hayes MP. Low-cost active electromyography. HardwareX. 2019;6.	1	1	$112	n				
Bravo-Martinez J. Open source automated western blot processor. HardwareX. 2019;6.	1	1	$135	n				
Utter B, Marbaker R, Eschen K, Abel J. Open-source experimental setup for investigating the actuation behavior of active textiles. HardwareX. 2019;6.	1	1	$1,940	n				
Guver A, Fifita N, Milas P, Straker M, Guy M, Green K, et al. A low-cost and high-precision scanning electrochemical microscope built with open source tools. HardwareX. 2019;6.	1	1	$300	$10,000	97%	97%	97%	97%
Romero-Morales AI, O’Grady BJ, Balotin KM, Bellan LM, Lippmann ES, Gama V. Spin∞: an updated miniaturized spinning bioreactor design for the generation of human cerebral organoids from pluripotent stem cells. HardwareX. 2019;6.	1	0	$2,500	n				
Klar V, Pearce JM, Kärki P, Kuosmanen P. Ystruder: Open source multifunction extruder with sensing and monitoring capabilities. HardwareX. 2019;6.	1	1	$150	$3,000	95%	95%	95%	95%
Matheny AM, Marchetto P, Powell J, Rechner A, Chuah J-Y, McCormick E, et al. LEAF: Logger for ecological and atmospheric factors. HardwareX. 2019;6.	0	0	$1,300	$0				
Rotermund D, Ernst UA, Pawelzik KR. Open Hardware for neuro-prosthesis research: A study about a closed-loop multi-channel system for electrical surface stimulations and measurements. HardwareX. 2019;6.	0	0	n	n				
Price A. An apparatus for personalized atmospheric and flight data collection aboard high altitude weather balloons. HardwareX. 2019;6.	0	1	$54	$272	80%		80%	
Chan SHM, Loke LHL, Crickenberger S, Todd PA. Robonerite: A low-cost biomimetic temperature logger to monitor operative temperatures of a common gastropod (Nerita spp.) in tropical urban seascapes. HardwareX. 2019;6.	0	0	$104	$257	60%			
Jo W, Hoashi Y, Paredes Aguilar LL, Postigo-Malaga M, Garcia-Bravo JM, Min B-C. A low-cost and small USV platform for water quality monitoring. HardwareX. 2019;6.	1	1	$201	n				
Alves-Oliveira P, Arriaga P, Paiva A, Hoffman G. Guide to build YOLO, a creativity-stimulating robot for children. HardwareX. 2019;6.	1	0	$200	$20,000	99%	99%		
Hill AP, Prince P, Snaddon JL, Doncaster CP, Rogers A. AudioMoth: A low-cost acoustic device for monitoring biodiversity and the environment. HardwareX. 2019;6.	0	0	$50	$50				
Yensen N, Allen PB. Open source all-iron battery for renewable energy storage. HardwareX. 2019;6.	0	0	$300	n				
Camprodon G, González Ó, Barberán V, Pérez M, Smári V, de Heras MÁ, et al. Smart Citizen Kit and Station: An open environmental monitoring system for citizen participation and scientific experimentation. HardwareX. 2019;6.	1	1	$995	n				
Vaut L, Scarano E, Tosello G, Boisen A. Fully replicable and automated retention measurement setup for characterization of bio-adhesion. HardwareX. 2019;6.	1	1	$500	n				
Bessler N, Ogiermann D, Buchholz M−B, Santel A, Heidenreich J, Ahmmed R, et al. Nydus One Syringe Extruder (NOSE): A Prusa i3 3D printer conversion for bioprinting applications utilizing the FRESH-method. HardwareX. 2019;6.	1	0	$100	n				
Jo Heuschele D, Wiersma J, Reynolds L, Mangin A, Lawley Y, Marchetto P. The Stalker: An open source force meter for rapid stalk strength phenotyping. HardwareX. 2019;6.	0	1	$300	n				
Alhaddad AY, Cabibihan J-J, Hayek A, Bonarini A. A low-cost test rig for impact experiments on a dummy head. HardwareX. 2019;6.	1	0	$3,795	n				
Spinelli GM, Gottesman ZL. A low-cost Arduino-based datalogger with cellular modem and FTP communication for irrigation water use monitoring to enable access to CropManage. HardwareX. 2019;6.	0	1	$400	$2,000	80%		80%	
Williams J, Mikhelson I. Triple frame buffer FPGA implementation. HardwareX. 2019;5.	0	0	$735	n				
Shaid A, Wang L, Padhye R, Gregory M. Low cost bench scale apparatus for measuring the thermal resistance of multilayered textile fabric against radiative and contact heat transfer. HardwareX. 2019;5.	0	1	$818	n				
Kurata K, Sumida K, Takamatsu H. Open-source cell extension system assembled from laser-cut plates. HardwareX. 2019;5.	0	0	$629	$6,290	90%			
Watson C, Senyo S. All-in-one automated microfluidics control system. HardwareX. 2019;5.	1	1	$1,730	$10,000	83%	83%	83%	83%
Wang B, Sud R, Leung M, Yang M, Rodriguez JA, Lee R, et al. OpenEM – Electromagnetic field mapping robot for microwave and RF measurements. HardwareX. 2019;5.	1	1	$750	n				
Frie JA, Khokhar JY. An open source automated two-bottle choice test apparatus for rats. HardwareX. 2019;5.	1	1	$136	n				
Montoya RÁ, Delgado S, Castilla J, Navarrete J, Contreras ND, Marijuan JR, et al. Methods to simplify cooling of liquid Helium cryostats. HardwareX. 2019;5.	0	0	$30	n				
Ulrich B. Open-source wideband (DC to MHz range) isolated current sensor. HardwareX. 2019;5.	0	0	$39	$3,823	99%			
Carlson DF, Fürsterling A, Vesterled L, Skovby M, Pedersen SS, Melvad C, et al. An affordable and portable autonomous surface vehicle with obstacle avoidance for coastal ocean monitoring. HardwareX. 2019;5.	1	0	$3,315	n				
Mariola M, Bemont C, Petruccione F. A novel analogue keyboard for embedded applications, based on integer division truncation. HardwareX. 2019;5.	0	1	$5	n				
Medina DAV, Rodriguez Cabal LF, Lanças FM, Santos-Neto ÁJ. Sample treatment platform for automated integration of microextraction techniques and liquid chromatography analysis. HardwareX. 2019;5.	0	1	$715	n				
Bhandare A, Patnaik A, Pommerenke D, Sharma S, Fischer D. Low cost fast frequency switching driver for Acousto-Optic Modulators used in laser cooling. HardwareX. 2019;5.	0	0	$900	$2,000	55%			
Allwright M, Zhu W, Dorigo M. An open-source multi-robot construction system. HardwareX. 2019;5.	1	1	$40,337	n				
Kitchener BGB, Dixon SD, Howarth KO, Parsons AJ, Wainwright J, Bateman MD, et al. A low-cost bench-top research device for turbidity measurement by radially distributed illumination intensity sensing at multiple wavelengths. HardwareX. 2019;5.	1	1	$581	$25,815	98%	98%	98%	98%
Robke R, Hashemi P, Ramsson E. A simplified LED-driven switch for fast-scan controlled-adsorption voltammetry instrumentation. HardwareX. 2019;5.	0	0	$7	n				
Netto GT, Arigony-Neto J. Open-source Automatic Weather Station and Electronic Ablation Station for measuring the impacts of climate change on glaciers. HardwareX. 2019;5.	1	1	$850	$15,000	94%	94%	94%	94%
Guillardi H Júnior, Liberado EV, Pomilio JA, Marafão FP. General-compensation-purpose Static var Compensator prototype. HardwareX. 2019;5.	0	0	$6,799	n				
Agcayazi T, Foster M, Kausche H, Gordon M, Bozkurt A. Multi-axis stress sensor characterization and testing platform. HardwareX. 2019;5.	1	1	$5,800	n				
Kumbol VW-A, Ampofo EK, Twumasi MA. Actifield, an automated open source actimeter for rodents. HardwareX. 2018;4.	1	1	$123	$6,150	98%	98%	98%	98%
Bentancor M, Vidal S. Programmable and low-cost ultraviolet room disinfection device. HardwareX. 2018;4.	0	1	$176	$1,000	82%		82%	
Lei T, Mohamed AA, Claudel C. An IMU-based traffic and road condition monitoring system. HardwareX. 2018;4.	0	0	$55	n				
Kumar Jha R, Srivastav Y, Sumbli V, Trisha, Gandhi V, Jain S. RFID based food rationing system. HardwareX. 2018;4.	0	1	$37	n				
Oberloier S, Pearce JM. Open source low-cost power monitoring system. HardwareX. 2018;4.	0	1	$155	$400	61%		61%	
Hietanen I, Heikkinen ITS, Savin H, Pearce JM. Approaches to open source 3-D printable probe positioners and micromanipulators for probe stations. HardwareX. 2018;4.	1	0	$145	n				
Lund J, Paris A, Brock J. Mouthguard-based wireless high-bandwidth helmet-mounted inertial measurement system. HardwareX. 2018;4.	1	1	$661	n				
Carvalho MC, Sanders CJ, Holloway C. Auto-HPGe, an autosampler for gamma-ray spectroscopy using high-purity germanium (HPGe) detectors and heavy shields. HardwareX. 2018;4.	1	1	$750	n				
LeSuer RJ, Osgood KL, Stelnicki KE, Mendez JL. OMIS: The Open Millifluidic Inquiry System for small scale chemical synthesis and analysis. HardwareX. 2018;4.	1	1	$103	n				
Schlatter S, Illenberger P, Rosset S. Peta-pico-Voltron: An open-source high voltage power supply. HardwareX. 2018;4.	0	1	$420	$7,700	95%		95%	
Slocum RK, Adams RK, Buker K, Hurwitz DS, Mason HB, Parrish CE, et al. Response spectrum devices for active learning in earthquake engineering education. HardwareX. 2018;4.	0	1	$265	n				
Kassis T, Perez PM, Yang CJW, Soenksen LR, Trumper DL, Griffith LG. PiFlow: A biocompatible low-cost programmable dynamic flow pumping system utilizing a Raspberry Pi Zero and commercial piezoelectric pumps. HardwareX. 2018;4.	1	0	$350	n				
Bellon JA, Pino MJ, Wilke N. Low-cost equipment for electroformation of Giant Unilamellar Vesicles. HardwareX. 2018;4.	1	0	$75	n				
Carrillo-Bucio JL, Tena-Garcia JR, Armenta-Garcia EP, Hernandez-Silva O, Cabañas-Moreno JG, Suárez-Alcántara K. Low-cost Sieverts-type apparatus for the study of hydriding/dehydriding reactions. HardwareX. 2018;4.	0	0	$10,000	n				
Raymond MA, Mast TG, Breza JM. An open-source lickometer and microstructure analysis program. HardwareX. 2018;4.	1	0	$216	n				
Gaudenzi Asinelli M, Serra Serra M, Molera Marimòn J, Serra Espaulella J. The smARTS_Museum_V1: An open hardware device for remote monitoring of Cultural Heritage indoor environments. HardwareX. 2018;4.	1	1	$24	n				
Ibarra D, Ledesma R, Lopez E. Design and construction of an omnidirectional sound source with inverse filtering approach for optimization. HardwareX. 2018;4.	1	0	$190	n				
Garcia VE, Liu J, DeRisi JL. Low-cost touchscreen driven programmable dual syringe pump for life science applications. HardwareX. 2018;4.	1	0	$603	$1,500	60%	60%		
Drackley B, Holtz M, Yang J. An inexpensive modified weight-bearing device assembled in-house for high throughput unbiased behavioral pain assessment in mice. HardwareX. 2018;4.	0	1	$725	n				
Woern AL, McCaslin JR, Pringle AM, Pearce JM. RepRapable Recyclebot: Open source 3-D printable extruder for converting plastic to 3-D printing filament. HardwareX. 2018;4.	1	1	$671	$6,000	89%	89%	89%	89%
Reinecke T, Clowers BH. Implementation of a flexible, open-source platform for ion mobility spectrometry. HardwareX. 2018;4.	0	0	$210	n				
Susko AQ, Gilbertson F, Heuschele DJ, Smith K, Marchetto P. An automatable, field camera track system for phenotyping crop lodging and crop movement. HardwareX. 2018;4.	0	0	$5,550	n				
Thompson AL, Conrad A, Conley MM, Shrock H, Taft B, Miksch C, et al. Professor: A motorized field-based phenotyping cart. HardwareX. 2018;4.	0	0	$4,000	n				
Fleming J, Amietszajew T, McTurk E, Greenwood D, Bhagat R. Development and evaluation of in-situ instrumentation for cylindrical Li-ion cells using fibre optic sensors. HardwareX. 2018;3:100–9.	0	0	$148	n				
White JA, Streets AM. Controller for microfluidic large-scale integration. HardwareX. 2018;3:135–45.	0	1	$200	n				
Carvalho MC, Murray RH. Osmar, the open-source microsyringe autosampler. HardwareX. 2018;3:10–38.	1	1	$477	$30,000	98%	98%	98%	98%
Pusch K, Hinton TJ, Feinberg AW. Large volume syringe pump extruder for desktop 3D printers. HardwareX. 2018;3:49–61.	1	0	$49	$200,000	100%	100%		
Winters BJ, Shepler D. 3D printable optomechanical cage system with enclosure. HardwareX. 2018;3:62–81.	1	0	$379	n				
Dellal D, Yee E, Lathwal S, Sikes H, Gomez-Marquez J. Low-cost plug and play photochemistry reactor. HardwareX. 2018;3:1–9.	1	1	$68	$6,800	99%	99%	99%	99%
Cain PW, Cross MD. An open-source hardware GPS data logger for wildlife radio-telemetry studies: A case study using Eastern box turtles. HardwareX. 2018;3:82–90.	0	1	$40	n				
Potticary J, Avery MP, Mills D, Hall SR. DONALD: A 2.5 T wide sample space permanent magnet. HardwareX. 2018;3:39–48.	0	0	$829	n				
Bravo-Martinez J. Open source 3D-printed 1000 μL micropump. HardwareX. 2018;3:110–6.	1	0	$43	n				
Liardon J-L, Hostettler L, Zulliger L, Kangur K, Gujja Shaik NS, Barry DA. Lake imaging and monitoring aerial drone. HardwareX. 2018;3:146–59.	1	1	$2,203	n				
Brower K, Puccinelli RR, Markin CJ, Shimko TC, Longwell SA, Cruz B, et al. An open-source, programmable pneumatic setup for operation and automated control of single- and multi-layer microfluidic devices. HardwareX. 2018;3:117–34.	1	0	$2,101	$21,010	90%	90%		
Kodera T. Adaptive antenna system by ESP32-PICO-D4 and its application to web radio system. HardwareX. 2018;3:91–9.	0	0	$29	n				
Dobbelaere T, Vereecken PM, Detavernier C. A USB-controlled potentiostat/galvanostat for thin-film battery characterization. HardwareX. 2017;2:34–49.	0	0	$100	$20,000	100%			
Chen X, Leon-Salas WD, Zigon T, Ready DF, Weake VM. A programmable optical stimulator for the Drosophila eye. HardwareX. 2017;2:13–33.	1	0	$757	n				
Liardon J-L, Barry DA. Adaptable imaging package for remote vehicles. HardwareX. 2017;2:1–12.	1	0	$441	n				
Ferretti J, Di Pietro L, De Maria C. Open-source automated external defibrillator. HardwareX. 2017;2:61–70.	0	0	$441	n				
Lupetti ML. Shybo. An open-source low-anthropomorphic robot for children. HardwareX. 2017;2:50–60.	1	1	$80	n				
Irgens P, Bader C, Lé T, Saxena D, Ababei C. An efficient and cost effective FPGA based implementation of the Viola-Jones face detection algorithm. HardwareX. 2017;1:68–75.	0	0	$310	n				
Chamorro-Posada P, Vázquez-Cabo J, Rodríguez JL, López-Santos JM. A plug’n’play WiFi surface-mount dual-loop antenna. HardwareX. 2017;1:46–53.	0	0	$13	n				
Dhankani KC, Pearce JM. Open source laboratory sample rotator mixer and shaker. HardwareX. 2017;1:1–12.	1	0	$30	$420	93%	93%		
Jiang J, Claudel C. A high performance, low power computational platform for complex sensing operations in smart cities. HardwareX. 2017;1:22–37.	0	0	$235	n				
Pocero L, Amaxilatis D, Mylonas G, Chatzigiannakis I. Open source IoT meter devices for smart and energy-efficient school buildings. HardwareX. 2017;1:54–67.	0	1	$248	n				
Oh J, Hofer R, Fitch WT. An open source automatic feeder for animal experiments. HardwareX. 2017;1:13–21.	1	1	$220	n				
McMunn MS. A time-sorting pitfall trap and temperature datalogger for the sampling of surface-active arthropods. HardwareX. 2017;1:38–45.	1	1	$215	n				
García-Pinillos F, Latorre-Román PÁ, Soto-Hermoso VM, Párraga-Montilla JA, Pantoja-Vallejo A, Ramírez-Campillo R, et al. Agreement between the spatiotemporal gait parameters from two different wearable devices and high-speed video analysis. PLOS ONE. 2019 Sep 24;14(9):e0222872.	0	0	n	n				
SignalBuddy [Internet]. OpenBehavior. 2019 [cited 2020 Jan 15]. Available from: https://edspace.american.edu/openbehavior/2019/09/19/signalbuddy/	1	1	$15.00	n				
Yallapragada VVB, Gowda U, Wong D, O’Faolain L, Tangney M, Devarapu GCR. ODX: A Fitness Tracker-Based Device for Continuous Bacterial Growth Monitoring. Anal Chem. 2019 Oct 1;91(19):12329–35.	1	1	$25.00	n				
Kang HJ, Yang J, Chun BJ, Jang H, Kim BS, Kim Y-J, et al. Free-space transfer of comb-rooted optical frequencies over an 18 km open-air link. Nature Communications. 2019 Sep 30;10(1):1–8.	0	0	n	n				
Törnbom K, Lundälv J, Palstam A, Sunnerhagen KS. “My life after stroke through a camera lens”- A photovoice study on participation in Sweden. PLOS ONE. 2019 Sep 11;14(9):e0222099.	0	0	n	n				
Kalwa U, Legner C, Wlezien E, Tylka G, Pandey S. New methods of removing debris and high-throughput counting of cyst nematode eggs extracted from field soil. PLOS ONE. 2019 Oct 15;14(10):e0223386.	1	0	$100.00	n				
Bernard C. Open Source Tools and Methods: A New Category of Short Papers to Share Knowledge, Accelerate Research, and Acknowledge Those Who Develop Such Tools and Methods. eNeuro [Internet]. 2019 Sep 1 [cited 2020 Jan 15];6(5). Available from: https://www.eneuro.org/content/6/5/ENEURO.0342–19.2019	0	0	n	n				
Scholz A, Eggenhofer F, Gelhausen R, Grüning B, Zarnack K, Brüne B, et al. uORF-Tools—Workflow for the determination of translation-regulatory upstream open reading frames. PLOS ONE. 2019 Sep 12;14(9):e0222459.	0	0	n	n				
Blin G, Sadurska D, Migueles RP, Chen N, Watson JA, Lowell S. Nessys: A new set of tools for the automated detection of nuclei within intact tissues and dense 3D cultures. PLOS Biology. 2019;17(8):e3000388.	0	0	n	n				
Gleeson P, Cantarelli M, Marin B, Quintana A, Earnshaw M, Sadeh S, et al. Open Source Brain: A Collaborative Resource for Visualizing, Analyzing, Simulating, and Developing Standardized Models of Neurons and Circuits. Neuron. 2019 Aug 7;103(3):395–411.e5.	0	0	n	n				
Morrison TJ, Sefton E, Marquez-Chin M, Popovic MR, Morshead CM, Naguib HE. A 3D Printed Device for Low Cost Neural Stimulation in Mice. Front Neurosci [Internet]. 2019 [cited 2020 Jan 15];13. Available: https://www.frontiersin.org/articles/10.3389/fnins.2019.00784/full	1	0	$1.00	n				
Singh S, Bermudez-Contreras E, Nazari M, Sutherland RJ, Mohajerani MH. Low-cost solution for rodent home-cage behaviour monitoring. PLOS ONE. 2019 août;14(8):e0220751.	0	0	$35.00	n				
Byagathvalli G, Pomerantz A, Sinha S, Standeven J, Bhamla MS. A 3D-printed hand-powered centrifuge for molecular biology. PLOS Biology. 2019 mai;17(5):e3000251.	1	0	$1.00	n				
Aidukas T, Eckert R, Harvey AR, Waller L, Konda PC. Low-cost, sub-micron resolution, wide-field computational microscopy using opensource hardware. Scientific Reports. 2019 May 15;9(1):1–12.	1	0	$150.00	n				
Colville MJ, Park S, Zipfel WR, Paszek MJ. High-speed device synchronization in optical microscopy with an open-source hardware control platform. Scientific Reports. 2019 Aug 21;9(1):1–13.	0	0	$525.00	$745.00	30%			
Kallmyer NE, Shin HJ, Brem EA, Israelsen WJ, Reuel NF. Nesting box imager: Contact-free, real-time measurement of activity, surface body temperature, and respiratory rate applied to hibernating mouse models. PLOS Biology. 2019 juil;17(7):e3000406.	0	1	$400.00	$5,000.00	92%		92%	
Chiapello M, Das D, Gutjahr C. Ramf: An Open-Source R Package for Statistical Analysis and Display of Quantitative Root Colonization by Arbuscular Mycorrhiza Fungi. Front Plant Sci [Internet]. 2019 [cited 2020 Jan 15];10. Available from: https://www.frontiersin.org/articles/10.3389/fpls.2019.01184/full	0	0	n	$600.00				
UV Transilluminators and Open source DIY kit — UV Transilluminator Manual [Internet]. [cited 2020 Jan 15]. Available from: http://public.iorodeo.com/docs/uv_transilluminator/on the Cheap	0	0	$225.00	$1,100.00	80%			
Pereira VR, Hosker BS. Low-cost (<€5), open-source, potential alternative to commercial spectrophotometers. PLOS Biology. 2019 juin;17(6):e3000321.	1	0	$6.00	$1,113.00	99%	100%		
Amann S, Witzleben M von, Breuer S. 3D-printable portable open-source platform for low-cost lens-less holographic cellular imaging. Scientific Reports. 2019 Aug 2;9(1):1–10.	1	0	$190.00	$900.00	79%	79%		
Godwin LW-, Brown D, Livingston R, Webb T, Karriem L, Graugnard E, et al. Open-source automated chemical vapor deposition system for the production of two- dimensional nanomaterials. PLOS ONE. 2019 Jan 16;14(1):e0210817.	0	1	$30,000.00	$95,000.00	68%		68%	
Portnova AA, Mukherjee G, Peters KM, Yamane A, Steele KM. Design of a 3D-printed, open-source wrist-driven orthosis for individuals with spinal cord injury. PLOS ONE. 2018 févr;13(2):e0193106.	1	0	$15.00	$140.00	89%	89%		
Nuñez I, Matute T, Herrera R, Keymer J, Marzullo T, Rudge T, et al. Low cost and open source multi-fluorescence imaging system for teaching and research in biology and bioengineering. PLOS ONE. 2017 Nov 15;12(11):e0187163.	1	0	$250.00	$10,000.00	98%	98%		
Forman CJ, Tomes H, Mbobo B, Burman RJ, Jacobs M, Baden T, et al. Openspritzer: an open hardware pressure ejection system for reliably delivering picolitre volumes. Scientific Reports. 2017 May 19;7(1):1–11.	1	1	$484.00	$2,691.00	82%	82%	82%	82%
Vera RH, Schwan E, Fatsis-Kavalopoulos N, Kreuger J. A Modular and Affordable Time-Lapse Imaging and Incubation System Based on 3D-Printed Parts, a Smartphone, and Off-The-Shelf Electronics. PLOS ONE. 2016 déc;11(12):e0167583.	1	1	$277.00	$5,000.00	94%	94%	94%	94%
Kinstlinger IS, Bastian A, Paulsen SJ, Hwang DH, Ta AH, Yalacki DR, et al. Open-Source Selective Laser Sintering (OpenSLS) of Nylon and Biocompatible Polycaprolactone. PLOS ONE. 2016 févr;11(2):e0147399.	1	1	$10,000.00	$400,000.00	98%	98%	98%	98%
Wittbrodt BT, Squires DA, Walbeck J, Campbell E, Campbell WH, Pearce JM. Open-Source Photometric System for Enzymatic Nitrate Quantification. PLOS ONE. 2015 août;10(8):e0134989.	1	0	$65.00	$433.00	85%	85%		
Rosenegger DG, Tran CHT, LeDue J, Zhou N, Gordon GR. A High Performance, Cost-Effective, Open-Source Microscope for Scanning Two-Photon Microscopy that Is Modular and Readily Adaptable. PLOS ONE. 2014 Oct 21;9(10):e110475.	1	0	n	n				
Shlyonsky V, Dupuis F, Gall D. The OpenPicoAmp: An Open-Source Planar Lipid Bilayer Amplifier for Hands-On Learning of Neuroscience. PLOS ONE. 2014 Sep 24;9(9):e108097.	0	0	$223.00	$8,900.00	97%			
Patel SR, Ghose K, Eskandar EN. An Open Source 3-D Printed Modular Micro-Drive System for Acute Neurophysiology. PLOS ONE. 2014 avr;9(4):e94262.	1	1	$739.00	$30,000.00	98%	98%	98%	98%
Campbell RAA, Eifert RW, Turner GC. Openstage: A Low-Cost Motorized Microscope Stage with Sub-Micron Positioning Accuracy. PLOS ONE. 2014 févr;9(2):e88977.	0	1	$1,000.00	$10,000.00	90%		90%	
Rowe AA, Bonham AJ, White RJ, Zimmer MP, Yadgar RJ, Hobza TM, et al. CheapStat: An Open-Source, “Do-It-Yourself” Potentiostat for Analytical and Educational Applications. PLOS ONE. 2011 Sep 13;6(9):e23783.	0	0	$80.00	$1,000.00	92%			
Miller AR, Davis GL, Oden ZM, Razavi MR, Fateh A, Ghazanfari M, et al. Portable, Battery-Operated, Low-Cost, Bright Field and Fluorescence Microscope. PLOS ONE. 2010;5(8):e11890.	0	0	$240.00	$1,875.00	87%			

Percent savings, S, was calculated by:

(1) $$S=\frac{\left(P-O\right)}{P}\left(\%\right)$$

where P is the proprietary cost of a commercial system and O is the open source device cost in U.S. dollars, which is limited to the material costs. The proprietary scientific hardware may also include a warranty and is generally fully assembled, whereas the FOSH is assumed to be self-built. The limitations to this approach and how to integrate labor costs are discussed in detail in Section 4.2. In general, the values used for P and O were conservatively limited to a direct cost comparison. These values were provided by the authors of the manuscript. In some cases, there were multiple values of O or P and in these cases representative (as close to direct) comparisons were made.

In addition, the average S was also calculated for those open source hardware devices that used 3-D printing, that used Arduinos and that used both.

## 3 Results

**Fig. 1:**
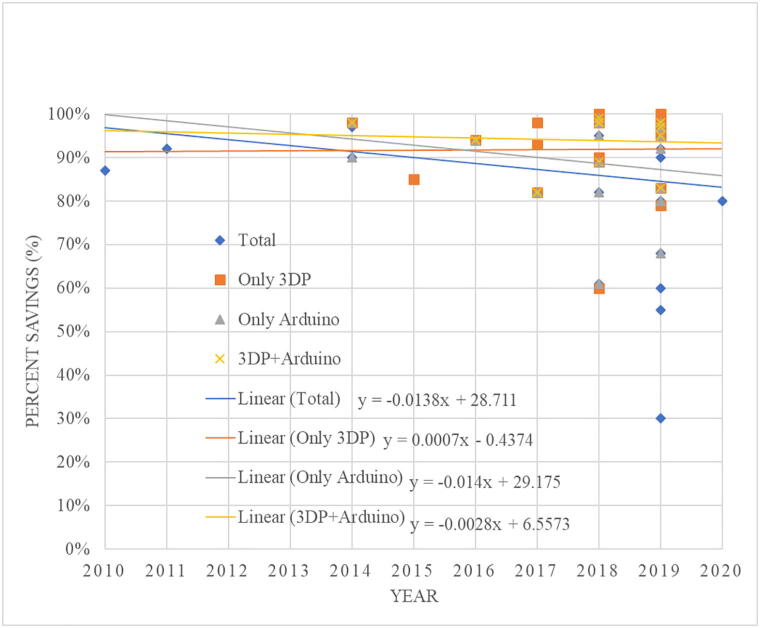
FOSH percent savings compared to proprietary tools as a function of year of publication.

The percent savings as a function of year of publication is shown in Fig. 1 for the FOSH that had percentage savings for 3-D printing only, Arduino only, the combination of both and those devices with only a total savings. As can be seen in Fig. 1 the spread in savings is becoming larger with time, although the vast majority of the published FOSH with savings that can be calculated are clustered over 90%. This clustering is more pronounced progressively with those that use Arduinos, 3-D printed devices, and the combination of the two technologies. The trend lines for the total and that use Arduinos are both negative with a slope of −0.014, which indicates that savings are becoming less over time. The same negative trend although less pronounced is seen for those FOSH that have both 3-D printing and Arduino technology (-0.0028). For the technologies that only use 3-D printing the slope is slightly positive (0.0007), which can be in part due to the reduction in 3-D printing filament costs over the same time period, however, these slopes must be used with caution. First, the number of technologies evaluated mean that the slightly positive slope seen for 3-D printing should be viewed statistically as roughly flat. Second, these values are in percents, which indicate a 1.4% drop in savings as compared to proprietary tools for the totals per year. Thus, linear forecasting into the future with a 95% confidence interval provides values of 80% cost savings in 2023 with the lower confidence bound of 68% and an upper confidence bound of 91%. This is, however, also misleading as can be seen by the data plotted in Fig. 1. For the three years when there are substantial open hardware that met the criteria for this study to evaluate for the amount of variation in percent savings (2017, 2018 and 2019) there is an increasing standard deviation of 7%, 13% and 19%, respectively. A single technology, providing a relatively meager 30% cost saving (high-speed device synchronization in optical microscopy) reduces the savings by 3% and increases the standard deviation by 5%. Evaluation of the results provides the conclusions that there is a clear expansion in the range of devices considered for open hardware development, which is broadening the potential savings to both 99% savings and higher as well as to lower percent savings.

As can be seen in both Table A1 and Fig. 1 the historical economic savings observed in the early open hardware literature are still present for newly developed technologies. The two supporting technologies that originally enabled widespread distributed manufacturing of open source scientific tools continue to play a significant role in open source scientific hardware with 3-D printing being used for 46% of the devices and Arduino microcontrollers being used for 39%. Thus, more than half of the devices (54%) did not use 3-D printing and an even greater number (61%) did not use Arduino technology. No other technologies, however, were found that were as widely used in the FOSH evaluated. Most of the open source technologies had economic costs calculated by the authors of the manuscripts (92.4%) although there were only proprietary cost equivalents for 37.8% of the devices. This difference is due to a combination of factors including both real novelty in some of the open hardware (e.g. there was no functionally equivalent commercial product) as well as many authors focusing on function over cost). For the scientific tools where the data was available, the average material costs from the aggregate of the bill of materials for the open hardware devices analyzed was under $1,500 and the average replacement cost for a proprietary device with equivalent functionality was well over $20,000. Overall the average open hardware scientific tool saved 87% compared to the proprietary tool. These savings ranged from on the high end over 99% for robots [21], sensors [22], reactors [23], analytical equipment [24] and digital manufacturing equipment [25]. The latter type of equipment that is used for the automation of experiments or further custom scientific experiments generally saved a large percentage over commercial products (e.g. laser sintering AM system [26] or an autosampler [27]). The open source hardware that saved the least (in terms of percentage) was from device synchronization [28] or on technologies that had wider application than science (e.g. it is mass produced for other applications like power monitoring [29]). These savings were still high with only one device under 50% savings and the standard deviation of only 15%. The representative example of the average open source hardware project was a portable, battery-operated, bright field and fluorescence microscope, which could be built for $240 and replaced a proprietary tool costing $1,875, thereby saving 87% [30].

These open source hardware economic values increased slightly to 89% for those that used Arduino technology and even more to 92% for those that used 3-D printing. It is well established that fabricating products with a distributed desktop 3-D printer result in lower costs for scientific equipment [1], [2], [4], [29], [31], [32], [33], [34], [35], [36], [37]. Similarly, automation with an Arduino is also known to reduce costs for a wide variety of experimental disciplines [1], [2], [4], [38], [39], [40], [41], [42], [43]. It should be noted that the use of the Arduino is not the lowest possible cost, as the microcontroller chips can be built into a custom PCB for less money than buying even the lower costs Arduinos (e.g. the nano or uno). However, the number of labs with ready access to the skills necessary to design and fabricate a board are limited (although there are several open source PCB mills available for low costs [44], [45]). The standard deviation also tightened for these classes of open hardware devices to 9% and 10% for 3-D printed and Arduino-using projects, respectively. These trends remained, but became even more substantial when the additive manufacturing and open source electronics technologies were combined. For open hardware devices that used both Arduino and 3-D printing the percent savings averaged 94% over commercial equivalents. The standard deviation shrunk to 6% although only about 10% of all open source hardware devices evaluated in Table A1 combined both of these techniques. Thus, the vast majority (90%) did not use both technologies.

## 4 Discussion

### 4.1 Limitations

This study had several limitations. First, the databases selected are far from a complete survey of all open source scientific hardware as it has grown to be quite substantial as evidence from Google Scholar results [3] as well as the list maintained on Appropedia (https://www.appropedia.org/Open-source_Lab). Last year (2019) alone, for example, records over 1,500 articles with the term “open source hardware” indexed by Google Scholar. However, both of the sources for articles were relatively recent (e.g. *HardwareX* started publishing in 2017) and both draw on hardware that would be of interest to a wide range of scientists unlike the specialty literature alone, which still makes up the majority of open hardware literature. These two sources also ensured that the hardware evaluated was indeed fully free and open source licensed. There could, however, be a selection bias present (e.g. the devices evaluated may haven been particularly amenable to open source approaches while this may not be the case for all scientific hardware).

Second, although the *HardwareX* articles almost all had a cost of the open hardware calculated as this is a requirement for the journal, many of the other articles did not. This means cost savings that do exist or were different from the average shown here were missed. In addition, many of the devices were for equipment that is not commercially available so direct apples-to-apples savings was not available. This is because one of the primary advantages of open source hardware is that it enables scientists to make new tools that enable new experiments and new science to be discovered. In addition, if a given study provided a range a wide range of values or had multiple comparisons, it was not used in the review calculations. So, for example, in the Hietanen et al.’s study [46] on three approaches to open source 3-D printable probe positioners and micromanipulators the different open source technologies were evaluated with a range of performances, costs (<$5 to $145 to make), and proprietary commercial equivalents. Analysis is complicated by the fact that the open source <$5 version is commercially available for $100. In addition, the simple open source probe holders that went on the positioners cost only $55 but replaced those that cost $450-$580 with a connector or $280-$305 with wire, or $490-$860 with a tri-axial connectors [46]. In addition, the shipping costs vary widely based on the country and the company for which the parts are being purchased. In many cases in the U.S. shipping is free, but in other countries VAT and other costs come into play. The resulting complexities from comparing all possible permutations and combinations for all the studies that had such complexities was beyond the scope of this article, although it is clear that in general distributed fabrication would save considerable sums of money depending on the labor costs.

Considerable future work is needed to evaluate FOSH in general and the specific FOSH evaluated here for is reliability and quality assurance of self-built scientific hardware. Much of the FOSH tools described had been validated for function, but not over the lifetime of the device. For a full direct comparison with proprietary scientific hardware future studies need to ascertain if self-built machines are prone to failure and errors at a different rate than their proprietary equivalents as well as the potential need for safeguards from fabrication errors to prevent known failure modes particularly if user or builder error could be the cause.

Lastly, the relevance of FOSH could be tracked in future work to evaluate the number of times a FOSH devices has been downloaded, replicated, forked, etc. using the analytics of osf.io and other repositories. This could provide the necessary data to calculate the total value to society and the return on investment for science funders following studies [9], [10], respectively.

### 4.2 Labor costs

The savings calculations used here assumed no labor cost, however, for the cases where labor that costs money is needed to fabricate the device, the time it takes to make it is simply multiplied by the labor cost including all overhead and benefits costs. These costs vary widely across the globe, type of institution and type of worker. Often the savings for the open hardware is so substantial that it is easy to justify in-house fabrication when the cost of the hardware is moderate to high. However, in some cases, it may be less costly to simply purchase a proprietary tool than to build it (e.g. when the worker is a highly-paid post doc at an elite Western university and the tool is complex and thus time consuming to make, but still has low commercial value because it is mass produced). To better help scientists make these decisions, ideally open hardware designers would always fabricate a second prototype using people external to the project to gain a better estimation of fabrication time for non-designers. It is likely that as open hardware becomes more common and scientists build off of past work, that knowing and reporting these times will become more common. Considerable future work is needed, however, in this area.

The cost savings determined assume that there is no labor cost for the purchasing of components, 3-D printing, and assembly of the free and open source scientific hardware. Most proprietary equipment comes fully assembled and often “plug and play” so it is important to analyze this assumption carefully to be able to compare the costs of the BOM of open hardware to that of commercial products. Zero labor costs are relevant in a number of academic situations that may represent a significant fraction of researchers. Thus, these cases will be analyzed in detail.

The fabrication of an open source hardware device, which has been previously designed, prototyped, tested and vetted (as the devices in Table A1 have been) are substantially easier to replicate than building one from scratch without plans. This is particularly true of the devices that rely on digital manufacturing techniques predominantly with the use of only a few “vitamins” (readily available commercial off-the-shelf components). Thus, the fabrication of equipment is often within reach of students at most levels. Let us consider the two foundational technologies as examples of this ease of replication: 3-D printing and Arduino.

3-D printing with a desktop 3-D printer, which makes up about half (46%) of the open source devices shown in Table A1 can be accomplished by low-skilled labor (e.g. anyone with basic computer literacy) [47]. In general, assuming a modern calibrated auto-bed leveling RepRap-class 3-D printer that used fused filament fabrication, the time investment for the printing process is a tiny fraction of the total print time. Depending on the printer, however, the maintenance and calibration of a 3-D printer can vary widely. For scientists that use their own systems the time investment to do this is necessary to include, but for others that use externally maintained systems (e.g. in libraries, machine shops, makerspaces, fab labs, etc.) the specific print time setup is representative of the time investment. The 3-D printer may need to run for hours to fabricate a part layer by layer from common plastics, but this can be accomplished untended by a human operator. The human 3-D printer operator’s time is truly limited as operation requires a “time investment” equivalent to approximately the cost of time for online shopping thanks to pre-made designs housed in free repositories. A relevant example would be the *NIH 3D Print Exchange* (3dprint.nih.gov), which provides models in formats that are readily compatible with RepRap 3-D printers and offers a custom labware category. It should be noted that the other major open design 3-D printing repositories (e.g. MyMiniFactory, YouMagine, Thingiverse, etc.) that offer a wide range (millions) of other products also contain substantial scientific hardware. Thus, instead of the lab worker inputting a purchase card or credit card information, the scientist would download the stl format file, slice following the predetermined settings and click print. For many open source 3-D printers there are also built-in presets for slicing (e.g. the quick print settings on a Lulzbot 3-D printer). As soon as the stl is loaded, the user clicks print and can then walk away and has no active participation in the manufacturing. For simple scientific equipment (e.g. a test tube rack) the material costs are extremely close to the total cost as the operating of a 3-D printer is barely influenced by the cost of electricity [48] as they are relatively efficient devices [49], [50]. There are also more complex fully 3-D printable devices that can print in place like a labjack or need only a modest amount of Lego-block like assembly (e.g. a centrifuge [51]). In general, it takes less than 1 min to load an stl, have it sliced in an open source slicing software like Cura of Slic3r, and click print (it should be noted, that large complex designs take longer to slice and may need many components printed separately).

Similar minimal time investments can also be seen for basic Arduino-based open source scientific hardware projects. The user follows what is often a simple wiring diagram for plugging components into the Arduino board. There are many “shields”, which are electric circuit boards that can be plugged on top of the Arduino PCB to extend its capabilities. The different shields, which can be found on the Arduino website and www.shieldlist.org follow the same philosophy as the original Arduino as they are easy to mount and can be produced for low-costs. There are shields for scientific applications ranging from pH monitoring [2] to medical devices [52] and measuring air quality using wireless self-powered devices [53]. Many of the studies reviewed in Table A1 either developed or used Arduino shields including references [23], [54], [55], [56], [57], [58], [59]. Fabrication of an Arduino device using a shield is often literally “plug and play” so again the time investment calculation is unnecessary. Although, it should be pointed out that this assumes a base level of technical sophistication as inexperienced people may not be able to follow wiring diagrams. This, experience could be for example gained either in makerspace-based Arduino workshops or by an undergraduate student finishing a controls class.

Many of the open hardware tools summarized in Table A1 do entail significant time for fabrication, assembly and calibration. Even for these devices at an educational institution the labor cost can still be zero. This can be the case when the fabrication of the equipment is used as part of training students as part of their educational experience. Students can, for example, fabricate their own lab equipment [60], [61], [62], [63], [64], [65]. In addition, the use of 3-D printing used in the classroom has been shown to not only save money but have several benefits related to empowering students [66], [67], [68]. As the tools they are fabricating are “research grade” they can bring students closer to doing actual science rather than simply copying recipes of former experiments.

Zero labor cost also holds true where the labor is provided by unpaid interns or volunteers. This can often be seen in universities, where for example undergraduates volunteer for research to gain experience and improve their resumes. Companies similarly sometimes have interns that are not being paid to gain experience. It should be pointed out here, that these zero labor cost situations describe the actual budget of a working scientist, which means that to obtain a given FOSH if scientists use these mechanisms their out of budget costs are only the materials costs.

Another relatively common zero labor cost situation is when there is no opportunity cost to using existing salaried employee (e.g., the use of a lab manager or RA, TA, or other position that is paid a fixed cost, and for which there is no opportunity cost for them working on the fabrication of the device). Many university employees have positions that do not involve continuous focused work during the times for which they are paid because their service is for example being accessible to answer student questions. These zero opportunity cost employees are already used for other tasks (e.g. photocopying exams) interspersed between their fixed time tasks (e.g. answering the phone). For example, a department secretary may be asked to use a 3-D printer to manufacturer dozens of parts for a complex scientific device as time allows between other duties the same as if this time were used to print out exam copies. Similarly, a PI with low discretionary funds may simply choose to use his own time to make a critical tool to gain enough preliminary data to be competitive on an external grant. Although the secretary or the PI is paid, there is no budget cost to their time from the perspective of an individual scientist’s budget.

### 4.3 Beyond savings to value

#### 4.3.1 Access to equipment

Although the results of this study showed that there were savings present calculating the benefits of FOSH for scientists when only considering savings are incomplete. First, the availability of low-cost FOSH makes some capabilities possible for researchers that would otherwise be prohibitively expensive or impossible. If a device would never have been purchased because for example it required winning an NSF Major Research Instrumentation (MRI) Program grant it is not appropriate to talk about savings. Only approximately a fifth of scientists that can meet the cost sharing and matching requirements under the Uniform Administrative Requirements, Cost Principles, and Audit Requirements for Federal Awards (Uniform Guidance) (2 CFR, Part 200) win an MRI in a given year. If a FOSH device costs only 30% of the proprietary device, all of the scientists that could obtain the match from their university could afford the equipment as cost sharing requirements for an MRI must be exactly 30% of the total project cost. With the availability of FOSH the entire scientific community becomes effectively wealthier even with no increase in budgets.

#### 4.3.2 Project management benefits of FOSH

The FOSH approach also provides substantial benefits from a project management standpoint for working scientists. With the rise of bureaucracies not aligned with discovery, administrative techniques can actively hamper science [69]. For example, it is not uncommon in academia to have the “types of money” not coincide with experimental reality. Mandatory complex budgets where every expenditure must be line-itemized and justified before purchase is now demanded by an increasing array of funders. It is time consuming and sometimes impossible to legally spend grant or contract funds for equipment if it has been budgeted to fund the salary of a student or staff member. This type of micro-managed budgeting restricts the freedom of the scientist to spend the money in the best way possible as often real science demands unpredictable expenditures several years out from a grant proposal budgeting exercise and “negotiation”. Open source hardware fabrication provides a relief valve for scientists trapped in these positions. For example, consider a scientist that won a grant to find a cure for a specific type of cancer. Most of her budget was slated for students to do lab work on existing equipment with a few thousand dollars for supplies and chemicals. Half way through the multi-year grant her team makes a promising new discovery, but needs high-throughput compartmentalization of many biological reactions to see it through. The perfect tool for this allows for automated control of multi-layer microfluidics and costs over $20,000. This is far more than her supplies budget and trading out research personnel time would not only ham-string her efforts by ruining group morale as she would need to fire a student researcher, but also may not be acceptable to her program manager. Fortunately, a programmable pneumatic setup for operation and automated control of single- and multi-layer microfluidic devices has been developed and open sourced [70]. She can use about $2,000 of her supplies budget to fabricate the equipment with her existing staff and move forward with the project that could benefit all of humanity.

#### 4.3.3 Non-Direct financial benefits to OSH for science

The results of this study clearly show there is a substantial economic benefit to using open source technologies as a scientist. There are other benefits that are secondary, which will be discussed here. These benefits could be converted into cost savings, but will be left for future work.

First, open source technology allows for many people to work on its development under some form of share-a-like license (e.g. GNU GPL v3), which is known to foster and accelerate innovation [71], [72], [73], [74]. This is because using open source provides a global community united around improving these technologies introduce new concepts and capabilities faster, better, and more effectively than internal teams working on proprietary solutions no matter how gifted the company or institution [71], [72], [73], [74]. In addition, development costs of future iterations of the technologies are lower as has been demonstrated by the free and open source software community [75], [76], [77]. This means that if a scientist adopts a particular tool today, they know that it is likely to be upgraded in the future in a way that they can benefit from (e.g. software or hardware on their machine with minimal additional charges because the license demands that those that make improvements re-share them with the community on the same license).

Second, proprietary instruments generally operate as “black boxes,” where scientists using them have access to only restricted information about the hardware and software’s internal workings [78]. In addition, these black boxes have spotty information for interfacing other instruments or software depending on the provider. An open source approach eliminates the black box as the technology, equations, and assumptions are all completely transparent. Scientists know exactly how a device works, which can provide more reliable research and insights into the phenomenon being studied.

Third, open source prevents vendor lock in and there is generally no push to upgrade. This has several benefits. Scientists are free to use other materials that do not come from the vendor of a particular instrument for example. This can reduce operating costs, but can also improve scientific flexibility and creativity. If scientists have the legal and technical ability to alter the code for hardware and software in their labs, they will never be left with stranded assets such as non-functioning equipment when commercial vendors go out of business, drop a product line, or looses key technical staff [79]. In addition, in cases when proprietary scientific tools no longer generates enough profit to justify their investment, companies can cripple their customers’ ability to do research if they stop producing or supporting a product. When a vendor for any reason stops supporting a product, that tool, which can be quite expensive, loses enormous value. This value is protected with open source technologies. In this way the open source nature of a product can be viewed as a form of insurance. With open source products the scientific community can always work to support or build upon it rather than discarding the technology or sunk assets.

Fourth, following closely on this benefit is the property of open source scientific technology to enable users to make extensive changes and complete customization of scientific equipment and software. This allows a much more complete control by the scientists of their research tools, which has benefits that drive high-quality science faster. The features of a given tool are based solely on merit instead of profit-based, which may appeal to the most users, but not a given specific user. As a given scientist has complete control over the equipment, he does not need to use or pay for features that he does not want or need. This can lead to higher quality for the specific researcher for a given tool.

Fifth, often open hardware can be fabricated faster than ordering it and waiting for shipping. This is particularly true for complex non-mass-produced tools with long lead times [1], [2].

Sixth, an open source approach still allows for commercialization using open source business models [79]. For example, consider a company that sells open source tools that are less expensive than proprietary offerings, while making their designs freely available. Their tools cost more than fabricating them yourself, but the small cost differential means that scientists would generally find it beneficial to purchase rather than make them. In this case, the scientist gets the best of both worlds – the freedom and control that comes from open source along with the ‘works out of the box’ method found in commercial offerings for only a relatively small fee.

Seventh, open source provides a culture of collaboration [80] that feeds well into learning communities of academia. With open source sharing of all research details it is easy for other scientists to follow one another’s work and thus build on it and cite it. Thus, open source technology is beneficial for tenure-track professors, as open source hardware can assist building professors’ tenure packages in all three areas of research, teaching, and service and can continue to bolster one’s academic careers after tenure. The strategic professor will open source valuable (from a research perspective) technologies to garner citations as others use it and cite it for years to come. The higher citations rates increase an academic’s h-index and thus opportunities for promotion, which has a direct economic impact on the individual faculty member in increased salary. In addition, universities, companies, or research groups that use open source methods can attract better talent. Open source culture also enable enterprises to access, recruit and retain top talent [81].

Finally, eighth as scientists are building their own equipment or using code they can scrutinize (or have other scrutinize), they benefit from increased security [82].

### 4.4 Policy implications

The economic costs of scientific equipment can not be ignored when trying to optimize the benefit of science for humanity or a specific nation. Even the wealthiest American scientists, who have dominated research expenditures for decades and have the most well-equipped research labs in the world [83], have limited access to best tools to do their work because of inflated prices of proprietary scientific equipment [2]. This lack of access slows the rate of scientific development in every field. In addition, the exorbitant costs of scientific instruments limit access to exciting and engaging labs in both K-12 and university education [84], which weakens recruitment into STEM (Science, Technology, Engineering and Math) fields and results in a drain on scientific talent for the future.

Free and open source methods not only offer the potential to radically reduce the cost of doing science, but also for training future scientists [85]. An entire university classroom of optics setups for a physics course can be printed in house for $500 using a selection of pre-designed components from the open-source optics library on a $250 open-source 3-D printer, replacing $15,000 of commercial equipment [16]. This would save over $66 million if scaled only to the basic physics labs in degree-granting institutions in the U.S. or over $500 million if scaled to all of the public and private secondary schools across America [86]. Obviously, the savings would mount to over $1b if scaled globally. The question appears no longer to be “Should we invest in open source hardware for science and education?” but rather “Is it economically responsible not to invest in open source technologies for science and education?”

It is clear there is an enormous return on investment (ROI) possible for those that fund both scientific research, but also STEM education by investing in free and open source technological development for the sciences.

To fully take advantage of this opportunity, nations must implement policies that allow knowledge to scale horizontally, leveraging open source methods. This horizontal scaling will be accomplished by national and state-level funding being spent only once for development of scientific equipment and then an immediate ROI is realized by the digital replication of the devices throughout the country for the costs of materials. For example, Moritz et al. quantified cost savings and as a result the value of an open source magnetic resonance imaging device (MRI) currently under development by the Open Source Imaging Initiative and found that depending on the scenario and the valuation method, savings for healthcare systems from US$1.8 million up to US$222 million per year are possible in the near future making the case for public funding and private investment in open source technology development [87]. There is a clear high ROI for medical open source technologies as well as for the sciences [88]. Similarly, Heikkinen, et al., found that by evaluating the research expenditures for a representative university, Finland alone could expect to save 2.84–27.7 m€ per year by strategic investment in open hardware development for technologies they spend the most funds purchasing [89]. Li et al. identified five economic motivations for FOSH companies, which can be applied to nations wanting to capitalize on the opportunity that open source affords: 1) reduce research and development costs as discussed above, 2) reduce recruiting costs using open source records of researcher skills, 3) eliminate patent intellectual property costs, 4) build a platform (e.g. Arduino) or 5) provide a related service (e.g. RedHat now owned by IBM) [90].

Open source technology is such a powerful tool within science because one researcher can create and publish a design, and all of us can benefit from it. This leads to an immediate opportunity to catch up to the best practices, so that it is possible for everyone in the field to continue to push science forward. By harnessing a scalable open-source methodology, funding is spent only once for development of scientific equipment and then a return on the investment is realized by direct digital replication of scientific devices for only the costs of materials. The return on investment is even more clear for FOSS where the cost of replication approaches zero dollars. Using this methodology will ensure that research-grade scientific instruments will be much more accessible at every level of the educational system and a greater percentage of the world’s scientists will be able to participate in experimental science. The ROI thus goes far beyond simply funding laboratories themselves as is well established, improvements in science lead to improvements in technology, which will enhance every aspect of the economy [91]. Historically these were on the order of 20–70% [91], but we know from the results of this study that the ROI for a single device goes beyond that if only copied by a single researcher. These open source scientific devices are being copied hundreds and thousands of times resulting in ROI for science funders ranging from 100 s to 1,000 s of percent after only a few months [10]. Future work is needed to track the ROI further than the first generation impacts of open source technologies (e.g. the wider effects on the economy). In addition, simply because high-quality FOSH designs are available does not mean that they will be adopted. Careful future analysis is needed to optimize the FOSH marketing to drive the highest possible ROI.

Given the results of this study it is clearly fiscally responsible to prioritize open source technologies for science research as in general the cost savings for FOSH are so substantial they enable tremendous effective labor values to be obtained. It is instructive to determine what the labor cost of a device would need to be to break even with a proprietary scientific tool. As an example, consider the 3-D printable open source desktop nutating mixer [6]. The overall cost in labor to source, print, and assemble it is about 1 h, which indicates that it is profitable for an organization to use the open source version if their labor costs are under $250/hour, even for the least expensive commercial equivalent (or under $330/hour for the average commercial system) [6].

To maximize the immediate potential of open source technology development in the sciences, nations can implement five policies:

1) Fund a study or develop a task force (e.g. like the National Academy of Sciences) to identify the top opportunities to realize strategic national goals and a high ROI for the creation of open-source scientific technologies (both software and hardware). The country’s largest current expenditures on scientific hardware should be determined along with the most likely future expenditures. All science-based equipment purchases from internationally-sourced suppliers can be ranked by value so equivalent (or superior) open-source technologies can be identified as either existing or needing to be developed as was done recently in Finland [89]. This enables prioritization of FOSH development for the scientific foci of a specific country or region (e.g. the European Union).
2) Fund the development of open source technology identified in 1). Where the potential FOSH savings are the most substantial, current funding for scientific hardware should be directed to FOSH projects and because of the high ROI of such projects further funding should be considered. The former will enable more scientists to utilize scientific hardware for the same costs and the latter may provide a much higher ROI than other expenditures.
3) Collect technology designs to create a national free on-line catalog of tested, vetted and validated free and open-source scientific tools, which would house the bill of materials, digital designs, instructions for assembly and operation and the source code for all software and firmware. Over time this vetted database can also include maintenance and reliability data to help scientists make the most informed decisions on their research equipment. Although, many scientific FOSH exist and are widely dispersed (e.g. having been downloaded thousands of times and presumably used) not all scientists are aware of them and one of the primary disadvantages is concerns over quality and validation. Such a database would provide this service as was seen with the NIH 3-D Print Exchange providing validated designs for both clinical and community use during the COVID-19 pandemic [92], [93], [94].
4) Institute, preferential purchasing guidelines for open source technologies particularly for validated tool sets from 3) for all government labs and all government funded projects similar to other federal programs meant to boost a specific technology (e.g. energy efficiency guidelines).
5) Finally, all policies should be revoked that are counter to open science, like those that discourage the use of open source technologies based on poor accounting practices [95]. Similarly, the institutional use of ‘preferred suppliers’, which are commercial in nature limits competition and directly limits purchasing FOSH or FOSH components. Lastly, government funding currently used to fund the development of proprietary scientific tools by any mechanism should be transferred to open source development of the functionally equivalent tool as it is highly likely to result in substantial cost savings as well as the other non-monetary benefits detailed here for the entire scientific enterprise.

## 5 Conclusions

The results of the review find overwhelming evidence for a wide range of scientific tools, that open source technologies provide substantial economic savings compared to equivalent or less functional proprietary tools. Overall and economic savings for the technologies reviewed was found to be 87% for using free and open source technologies. These economic savings increased slightly to 89% for those that used open source Arduino technology and even more to 92% for those that used RepRap-class 3-D printing. Combining both Arduino and 3-D printing, the savings averaged 94% over commercial equivalents. The results provide strong evidence for financial support of open source hardware and software development for the sciences. Given the overwhelming economic advantages of free and open source technologies, it appears financially responsible to divert funding proprietary scientific tools and most especially their development (e.g. SBIR/STTRs for proprietary devices in the U.S.) to the purchase and development of FOSH. Policies were outlined that provide nations with a template for strategically harvesting the opportunities provided by free and open source technological development.

## Declaration of Competing Interest

The authors declare that they have no known competing financial interests or personal relationships that could have appeared to influence the work reported in this paper.
